# Interplay between IL6 and CRIM1 in thiopurine intolerance due to hematological toxicity in leukemic patients with wild-type *NUDT15* and *TPMT*

**DOI:** 10.1038/s41598-021-88963-5

**Published:** 2021-05-06

**Authors:** Hyery Kim, Seungwon You, Yoomi Park, Jung Yoon Choi, Youngeun Ma, Kyung Tak Hong, Kyung-Nam Koh, Sunmin Yun, Kye Hwa Lee, Hee Young Shin, Suehyun Lee, Keon Hee Yoo, Ho Joon Im, Hyoung Jin Kang, Ju Han Kim

**Affiliations:** 1 Department of Pediatrics, Asan Medical Center Children’s Hospital, University of Ulsan College of Medicine, 88, Olympic-ro 43-gil, Songpa-gu, Seoul, 05505 Korea; 2 Seoul National University Biomedical Informatics (SNUBI), Division of Biomedical Informatics, Seoul National University College of Medicine, 101 Daehak-ro, Jongno-gu, Seoul, 03080 Korea; 3 Department of Pediatrics, Seoul National University College of Medicine, Seoul, 03080 Korea; 4 Seoul National University Cancer Research Institute, Seoul, Korea; 5 Department of Pediatrics, Seoul National University Bundang Hospital, Seoul, Korea; 6 Department of Information Medicine, Asan Medical Center and University of Ulsan College of Medicine, Seoul, 05505 Korea; 7 Department of Biomedical Informatics, College of Medicine, Konyang University, Taejon, Korea; 8 Department of Pediatrics, Samsung Medical Center, Sungkyunkwan University School of Medicine, Seoul, Korea

**Keywords:** Pharmacogenomics, Personalized medicine

## Abstract

*NUDT15* and *TPMT* variants are strong genetic determinants of thiopurine-induced hematological toxicity. Despite the impact of homozygous *CRIM1* on thiopurine toxicity, several patients with wild-type *NUDT15, TPMT,* and *CRIM1* experience thiopurine toxicity, therapeutic failure, and relapse of acute lymphoblastic leukemia (ALL). Novel pharmacogenetic interactions associated with thiopurine intolerance from hematological toxicities were investigated using whole-exome sequencing for last-cycle 6-mercaptopurine dose intensity percentages (DIP) tolerated by pediatric ALL patients (*N* = 320). *IL6* rs13306435 carriers (*N* = 19) exhibited significantly lower DIP (48.0 ± 27.3%) than non-carriers (*N* = 209, 69.9 ± 29.0%; *p* = 0.0016 and 0.0028 by *t* test and multiple linear regression, respectively). Among 19 carriers, 7 with both heterozygous *IL6* rs13306435 and *CRIM1* rs3821169 showed significantly decreased DIP (24.7 ± 8.9%) than those with *IL6* (*N* = 12, 61.6 ± 25.1%) or *CRIM1* (*N* = 94, 68.1 ± 28.4%) variants. *IL6* and *CRIM1* variants showed marked inter-ethnic variability. Four-gene-interplay models revealed the best odds ratio (8.06) and potential population impact [relative risk (5.73), population attributable fraction (58%), number needed to treat (3.67), and number needed to genotype (12.50)]. Interplay between *IL6* rs13306435 and *CRIM1* rs3821169 was suggested as an independent and/or additive genetic determinant of thiopurine intolerance beyond *NUDT15* and *TPMT* in pediatric ALL*.*

## Introduction

Despite improvements in combination drug therapy and risk stratification, approximately 20% of pediatric patients with acute lymphoblastic leukemia (ALL) still experience drug resistance and treatment failure due to drug toxicities. In European populations, about 50% of thiopurine-induced cytotoxic adverse reactions, such as severe neutropenia and leukopenia, are explained by *NUDT15* and *TPMT* genetic variants^1–4^. The Clinical Pharmacogenetics Implementation Consortium (CPIC)^5^ publishes practical guidelines for the implementation of pharmacogenetic (PGx) testing of thiopurine by using traditional star (*) allele-based molecular phenotyping for *NUDT15* and *TPMT*^6,7^.

According to the established guideline, the thiopurine dose is pharmacogenetically titrated based on the known risk variants of *NUDT15* and *TPMT*. However, a substantial proportion of patients with leukemia presenting no genetic variation in *NUDT15* or *TPMT* still experience life-threatening toxicities, which may result in dose reduction and/or discontinuation of thiopurine, resulting in therapeutic failure and relapse of leukemia. In an attempt to overcome the PGx gap, *CRIM1* rs3821169 homozygote has been identified in East Asians as a novel risk variant of thiopurine-induced hematological toxicities^8^. Heterozygotes of the variant have revealed only mild effect on thiopurine toxicity with an unknown clinical impact. However, its high prevalence (T = 0.066, Phase 3 of the 1000 Genomes Project^9^) and remarkable inter-ethnic variability (Table 2) might have severely confounded previous PGx studies assessing thiopurine toxicity. Therefore, investigating PGx interactions of novel genes/variants, other than *NUDT15* and *TPMT* variations, is urgently needed for preventing thiopurine intolerance due to hematological toxicities and improving pediatric ALL care.

The categorical nature of the traditional star allele haplotype-based method can complement the quantitative nature of gene-wise variant burden (GVB) method for evaluating the complex interplay of multiple genes/variants^10^. For instance, designating three categories [i.e., poor (PM), intermediate (IM), and normal (NM) metabolizers] per gene creates an exponentially increasing complexity of 3^*N*^ for a drug with *N*-gene PGx interactions. *NUDT15* and *TPMT* have been assigned nine PGx subgroups for thiopurine, which will increase exponentially following new PGx discoveries across different ethnic groups. GVB quantitates the cumulative variant burden of one or more genes into a single score with dimensionality reduction, thus providing a reliable frame for multiple gene-interaction analysis^11–13^.

In the present study, we aimed to identify novel PGx interactions associated with thiopurine toxicity in pediatric ALL patients carrying both wild-type (WT) *NUDT15* and *TPMT* (and not carrying homozygous *CRIM1* rs3821169) by using whole-exome sequencing (WES) technology. Our investigation of the effect of novel candidate PGx variants on the last-cycle 6-mercaptopurine (6-MP) dose intensity percentage (DIP) tolerated by pediatric patients with ALL, revealed clinically significant hematological toxicities and thiopurine intolerance. Our results provide not only the measures of clinical validity but also the measures of population impact (or clinical utility), including relative risk (RR), population attributable fraction (PAF), number needed to treat (NNT), and number needed to genotype (NNG)^14^, for preventing thiopurine toxicity.

## Methods

### Subjects

As described in our previous study, we recruited 320 Korean pediatric patients with ALL, who underwent maintenance therapy with 6-MP at three teaching hospitals, Seoul National University Hospital (SNUH), Asan Medical Center (AMC), and Samsung Seoul Medical Center (SMC), located in Seoul, South Korea. All subjects conformed with the exclusion criteria (i.e., relapse of the disease, stem cell transplantation, Burkitt’s lymphoma, mixed phenotype acute leukemia, infant ALL, or very high-risk of ALL)^8^. Patients were assigned to the standard-risk group if they were 1–9 years of age at the time of diagnosis with a white blood cell (WBC) count less than 50 × 10^9^/L; all other patients were assigned to the high-risk group. Patients underwent hematopoietic stem cell transplantation if they met one or more of the following criteria: age younger than 1 year, hypodiploidy, the presence of t(9;22), a WBC count equal to or greater than 200 × 10^9^/L, or the 11q23 rearrangement^15^. Patients allocated to the standard-risk group were treated with Children’s Cancer Group (CCG)-1891^16^, CCG-1952^17^ or Children’s Oncology Group (COG) AALL-0331 regimens^18^. In high-risk groups, CCG-1882^19^, 0601, or 1501 protocols for Korean multicenter studies^20^ were employed. In Korea, the planned dose of 6-MP was modified from 75 to 50 mg/m^2^, as several patients who had been administered the same dose under the original Western protocol exhibited moderate to severe toxicities during 6-MP administration^15,21^. The 6-MP doses during maintenance therapy were adjusted to maintain a WBC count of 2.0–3.5 × 10^9^/L, with an absolute neutrophil count (ANC) of over 500/μL. Hepatotoxicity-related dose modifications were primarily based on the COG guidelines; however, they were also performed at the discretion of the treating physician as this study was not undertaken per the uniform prospective protocols. Hematological toxicity as the clinical endpoint was estimated by the tolerated last-cycle 6-MP DIP (%). The percentage of the actual prescribed amount to the planned dose (50 mg/m^2^) was defined as the last-cycle 6-MP DIP using the recorded 6-MP dose per meter body surface area over the last-cycle (12-week) of maintenance. Doses employed for the last maintenance cycle were considered, as dose modification of 6-MP was mainly adopted during the early phase of maintenance. Further detailed descriptions of patients and measurements have been summarized in our previous study^8,15,21^. The present study was approved by the SNUH, AMC, and SMC Institutional Review Boards. Written informed consent was obtained from each participant. For the participants under the age of 18 years, informed consent was obtained from a parent and/or legal guardian. All experiments and methods were performed in accordance with the relevant guidelines and regulations.

### Whole-exome sequencing and pharmacogenomic subgrouping

**Figure 1:**
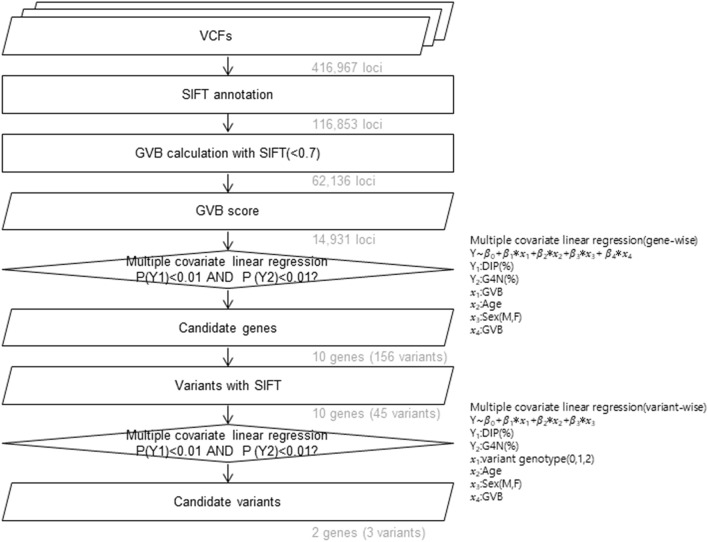
Workflow schema of the discovery phase. Variants with SIFT score less than 0.7 were incorporated into the GVB score calculation. Gene-wise multiple covariate linear regression was performed, and filtered genes were defined as candidate genes. Next, variant-wise multiple covariate linear regression was performed, and variants with filtered thresholds were deemed candidate variants. *SIFT* sorting intolerant from tolerant, *GVB* gene-wise variant burden.

WES data were obtained for pediatric patients with ALL patients and analyzed in a bioinformatics pipeline as previously described^8,10,11^. CPIC provides major PGx genes with haplotype definitions and molecular function annotations based on star (*) nomenclature. We classified patients with ALL into PM, IM, and NM groups for each gene, *NUDT15* and *TPMT*, according to CPIC classifications^6,7^. For both genes, we considered NMs as WTs. As in our previous study, data regarding DIP and the relative frequency of neutropenia (ANC < 500 μL) was available for the discovery cohort (N = 244)^8^. The relative frequency of neutropenia was defined by the ratio of frequencies of complete blood cell counts (CBC) with neutropenia from among the total tested CBCs. However, data regarding the frequency of neutropenia was not collected for the replication cohort (N = 76) at the time of this analysis. Therefore, we conducted a variant selection process using the discovery cohort. First, we performed multivariate linear regression, adjusting age, sex, and body surface area. Of 14,931 genes with GVB scores, 10 genes were identified with statistical significance at both DIP and the relative frequency of ANC < 500 μL. Next, we identified 45 variants with SIFT (sorting intolerant from tolerant) scores from among 156 variants of genes that passed multivariate linear regression with GVB score. Among the 45 variants, 3 variants passed the multivariate linear regression cutoff for SNPs^22^. Finally, we selected *IL6* rs13306435 as a novel candidate, which was only a missense variant among 3 variants (Fig. 1, Supplementary Table S1). We observed that no star name had been designated to the novel (or candidate) PGx genes. Thus, for the purpose of the present study, we defined non-carriers of *CRIM1* rs3821169 and *IL6* rs13306435 as WT carriers (WTs) of *CRIM1* and *IL6*, respectively (Table 2). Haplotypes were determined using PHASE 2.1.1^23,24^.

### Gene-wise variant burden for evaluating single- and multi-gene effects

GVB analysis was performed to evaluate the aggregated impact of both common and rare variants^10,11^. For each individual, the GVB of a coding gene was defined as the geometric mean of the SIFT^22^ scores of the coding variants (with SIFT score < 0.7) in the coding gene, where GVB^*G*^ denotes the GVB score of gene *G* [range 0.0–1.0]. The more deleterious the variant burden, the lower the score. First, we included *NUDT15* and *TPMT* in GVB analysis because these genes are clinically recognized to be related to thiopurine-induced toxiticy and have clinical guidelines like CPIC guidelines. Additionally, we included *CRIM1* rs3821169, which was identified in our previous study to define conditional GVB^8^. Finally, we included *IL6* rs13306435 following the selection process stated above. The multi-gene effect was evaluated by defining GVB^*A,B,C*^ as the geometric mean of GVB^*A*^, GVB^*B*^ and GVB^*C*^ [range 0.0–1.0]. Gene-variant interaction was considered by defining conditional GVB^*G^*(*variant*)^ as the GVB score of gene *G*, depending on the presence or absence of the specified *variant*. For example, GVB^*CRIM1^(rs13306435)*^ equals GVB^*CRIM1*^ when rs13306435 is present, vanishing to a WT score of 1.0 when absent.

### Inter-ethnic variability of allele frequencies and molecular phenotypes

Using the 2504 whole-genome sequences with multiple ethnicities provided by the 1000 Genomes Project phase 3^9^, we investigated inter-ethnic distributions of PGx alleles and haplotypes, along with their molecular phenotypes associated with thiopurine intolerance due to hematological toxicities (Table 2).

### Statistical analysis

The last-cycle 6-MP DIPs (%) according to different PGx groups were assessed using Student’s *t* test or one-way ANOVA with posthoc Tukey test. Multiple linear regression was also applied to adjust for confounding clinical variables. The powers of GVB^*NUDT15*^, GVB^*TPMT*^, GVB^*CRIM1*^, and GVB^*IL6*^, and their combinations for predicting 6-MP DIPs, were systematically evaluated by analyzing ROC (receiver operating characteristic) curves across eight different DIP cutoffs (i.e., 10%, 15%, 25%, 35%, 45%, 60%, 80%, and 100%) in terms of AUCs (areas under the ROC curves) (Figs. 3, 4). An ROC curve is a two-dimensional depiction of classification performance integrating all sensitivity and specificity values at all cutoff levels^25^. All statistical analyses were performed using the R statistical package (version 3.5.1). R package ‘pROC’ was used for calculating AUC values^26^. The optimal cutoff for the GVB score was determined by maximizing Youden’s index^27^.

GVB^*CRIM1^*(rs3821169*)^ was applied to control the potential confounding effect of the impressively high carrier frequency in East Asians [43.7% (= 220/504)], compared with other ethnicities (0.2–9.4%), and the mild effect of heterozygous expression on thiopurine intolerance attributed to hematological toxicities. GVB^*CRIM1^*(rs3821169*)^ denotes a conditional GVB score of *CRIM1* dependent on the presence or absence of homozygous rs3821169 variant (denoted as rs3821169*). It equals GVB^*CRIM1*^ when the subject carries homozygous *rs3821169* variant and otherwise vanishes to 1.0.

### Clinical validity parameters

We calculated and assessed clinical validity for each statistical parameter as follows.

Positive predictive value (PPV) implies the probability of an event when the genetic variant is present. In contrast, negative predictive value (NPV) means the probability of no event when the genetic variant is absent.

NNT is the inverse of the absolute of intervention, that is, the difference between the proportion of events in the control group and the proportion of events in the case group, which can be written as.

1 $$\frac{1}{({P}_{c}-{P}_{i})}.$$

If NNT is 20, it implies that 20 patients are needed to prevent an event like death or an adverse effect. NNG is the number of patients who must be genotyped to avoid one patient from experiencing an adverse event, which can be predicted based on following formula

2 $$\frac{NNT}{({P}_{c}+{P}_{i})}$$

For example, an NNG of 33 means that one adverse event was avoided for every 33 patients genotyped.

RR is the relative ratio of the proportion of events in the control group and the proportion of events in the case group, which is calculated by following formula.

3 $$\frac{{P}_{i}}{{P}_{c}}.$$

Odds ratio (OR) is calculated based on the comparison of the relative odds of an event in each group, which can be determined as

4 $$\frac{\frac{{P}_{i}}{(1- {P}_{i})}}{\frac{{P}_{c}}{(1- {P}_{c})}}.$$

In the above Eqs. (1),(2),(3),(4), ${P}_{c}$ is the proportion of events in the control group and ${P}_{i}$ is the proportion of events in the case group. PAF is the proportion of events that would be eliminated from the population if exposure to the risk factor were eliminated, which can be assessed as

5 $$\frac{P\left(Y=1\right)-P(Y=1|X=0)}{P(Y=1)}.$$

In Eq. (5), Y is an event development and X is a binary risk factor^14^.

## Results

### *IL6* rs13306435 as a novel pharmacogenetic variant for thiopurine intolerance due to hematological toxicities

We classified patients into three groups according to the variant status of *NUDT15* and *TPMT* to identify new variants not confounded by the two most critical PGx genes associated with thiopurine intolerance. Table 1 describes the clinical characteristics of 320 pediatric patients with ALL according to their PGx subgroups, presenting 80 patients who were non-WTs (i.e., IMs or PMs) of *NUDT15* and/or *TPMT* (*N* = 80), 115 patients who were all WTs (WT carriers of all the four genes), and 125 who were WTs of both genes, *NUDT15* and *TPMT* (both WTs) and carried *CRIM1* rs3821169 and/or *IL6* rs13306435 variants. Of the 125 patients with WT characterization for both *NUDT15* and *TPMT*, 94, 12, 11, and 8 patients belonged to the heterozygous *CRIM1*, heterozygous *IL6*, homozygous *CRIM1*, and *IL6* and *CRIM1* variant groups, respectively (Table 1).We used patients with all WTs (*N* = 115) as a control group for the following analysis. The average of the tolerated 6-MP DIPs of non-WTs for *NUDT15* (47.1 ± 30.5%, *N* = 72) and/or for *TPMT* (56.6 ± 33.6%, *N* = 9) were significantly lower than that of all WTs (71.3 ± 29.6%, *N* = 115) (*p* < 0.001, Table 1). The patients with homozygous *CRIM1* (dark blue circle in Fig. 3) tolerated significantly lower 6-MP DIP than the patients with all WTs before (*N* = 16, 44.6 ± 35.2%) or after (*N* = 11, 42.3 ± 35.0%) controlling the five subjects with *NUDT15* (59.76 ± 37.24%) or *IL6* (9.77%) variants.

**Table 1:** Clinical characteristics of 320 pediatric ALL patients with 6-MP maintenance therapy according to their pharmacogenetic subgroups of *NUDT15*, *TPMT*, *CRIM1,* and IL6 genes.

Characteristics	All WTs *N* = 115	NUDT15 or TPMT Non-WT *N* = 80^†^		NUDT15 and TPMT both WTs *N* = 125			
	WTs for all of *NUDT15*, *TPMT*, *CRIM1*, and *IL6*	*NUDT15* non-WT^†^	*TPMT* non-WT^†^	*CRIM1* rs3821169 heterozygote only	*IL6* rs13306435 heterozygote only	*CRIM1* rs3821169 homozygote only	*IL6* and *CRIM1* hetero- or homozygote
No. of subjects	115 (35.94%)	72 (22.50%)	9 (2.81%)	94 (39.17%)	12 (5.00%)	11 (4.38%)	8 (3.33%)
Age, median (range, year)	5.3 (1.2–19.4)	4.6 (1.7–15.8)	3.6 (1.3–14.8)	5.7 (1.1–17.0)	5.1 (2.4–17.0)	5.9 (2.8–21.8)	3.8 (1.7–7.9)
Sex							
Male	58	43	6	63	8	7	1
Female	57	29	3	31	4	4	7
Risk group							
Standard-risk	73	46	4	59	8	8	4
High-risk	42	26	5	35	4	3	4
**Last-cycle 6-MP dose intensity percentage, (%)**							
~ 10	2 (1.7%)	9 (12.5%)	0 (0.0%)	2 (2.1%)	1 (8.3%)	2 (18.2%)	1 (12.5%)
10–15	1 (0.9%)	3 (4.2%)	1 (11.1%)	1 (1.1%)	0 (0.0%)	1 (9.1%)	1 (12.5%)
15–25	4 (3.5%)	9 (12.5%)	1 (11.1%)	2 (2.1%)	0 (0.0%)	1 (9.1%)	2 (25.0%)
25–35	5 (4.4%)	7 (9.7%)	1 (11.1%)	9 (9.6%)	1 (8.3%)	2 (18.2%)	3 (37.5%)
35–45	5 (4.4%)	9 (12.5%)	1 (11.1%)	5 (5.3%)	0 (0.0%)	2 (18.2%)	1 (12.5%)
45–60	26 (22.6%)	11 (15.3%)	1 (11.1%)	16 (17.0%)	4 (33.3%)	0 (0.0%)	0 (0.0%)
60–80	31 (27.0%)	14 (19.4%)	2 (22.2%)	32 (34.0%)	2 (16.7%)	0 (0.0%)	0 (0.0%)
80–100	23 (20.0%)	7 (9.7%)	1 (11.1%)	13 (13.8%)	4 (33.3%)	3 (27.3%)	0 (0.0%)
100–	18 (15.7%)	3 (4.2%)	1 (11.1%)	14 (14.9%)	0 (0.0%)	0 (0.0%)	0 (0.0%)
Total	115 (100.0%)	72 (100.0%)	9 (100.0%)	94 (100.0%)	12 (100.0%)	11 (100.0%)	8 (100.0%)
Average ± SD (%)	71.31 ± 29.55	47.14 ± 30.48	56.56 ± 33.62	68.07 ± 28.39	61.59 ± 25.13	42.30 ± 34.97	22.86 ± 9.76

*ALL* acute lymphoblastic leukemia, *6-MP* 6-mercaptopurine, *WT* wild-type, *SD* standard deviation.

^†^One subject showed non-WT characterization for both *NUDT15* and *TPMT* genes. Values are the number of subjects (percentage) unless specified. Age means the age at the start of 6-MP maintenance therapy.

To rule out the PGx effect of *NUDT15*, *TPMT*, and homozygous *CRIM1* on thiopurine intolerance, we extracted 228 samples of non-carriers for these variants for the further discovery of novel PGx variants. We observed that carriers of *IL6* rs13306435 (*N* = 19, 48.0 ± 27.3%) exhibited significantly lower 6-MP DIPs than non-carriers (*N* = 209, 69.9 ± 29.0%), as evaluated by Student’s *t* test (*p* = 0.0016) and multiple covariate linear regression (*p* = 0.0028). Furthermore, of the 19 carriers, 7 patients with both *IL6* rs13306435 and *CRIM1* variants demonstrated significantly lower 6-MP intolerance, with a DIP of 24.7 ± 8.9% when compared with the DIP of 12 patients harboring only *IL6* rs13306435 variant (61.6 ± 25.1%; orange circle in Fig. 3). The potential interplay between *IL6* and *CRIM1* variants was suggested, which was further supported by the finding that seven patients with both *IL6* and *CRIM1* variants showed significantly lower 6-MP DIPs (24.7 ± 8.9%) than 94 heterozygous *CRIM1* carriers (68.1 ± 28.4%; light blue circle in Fig. 3).

### Interplay of *IL6* and *CRIM1* variants in thiopurine toxicity

**Figure 2:**
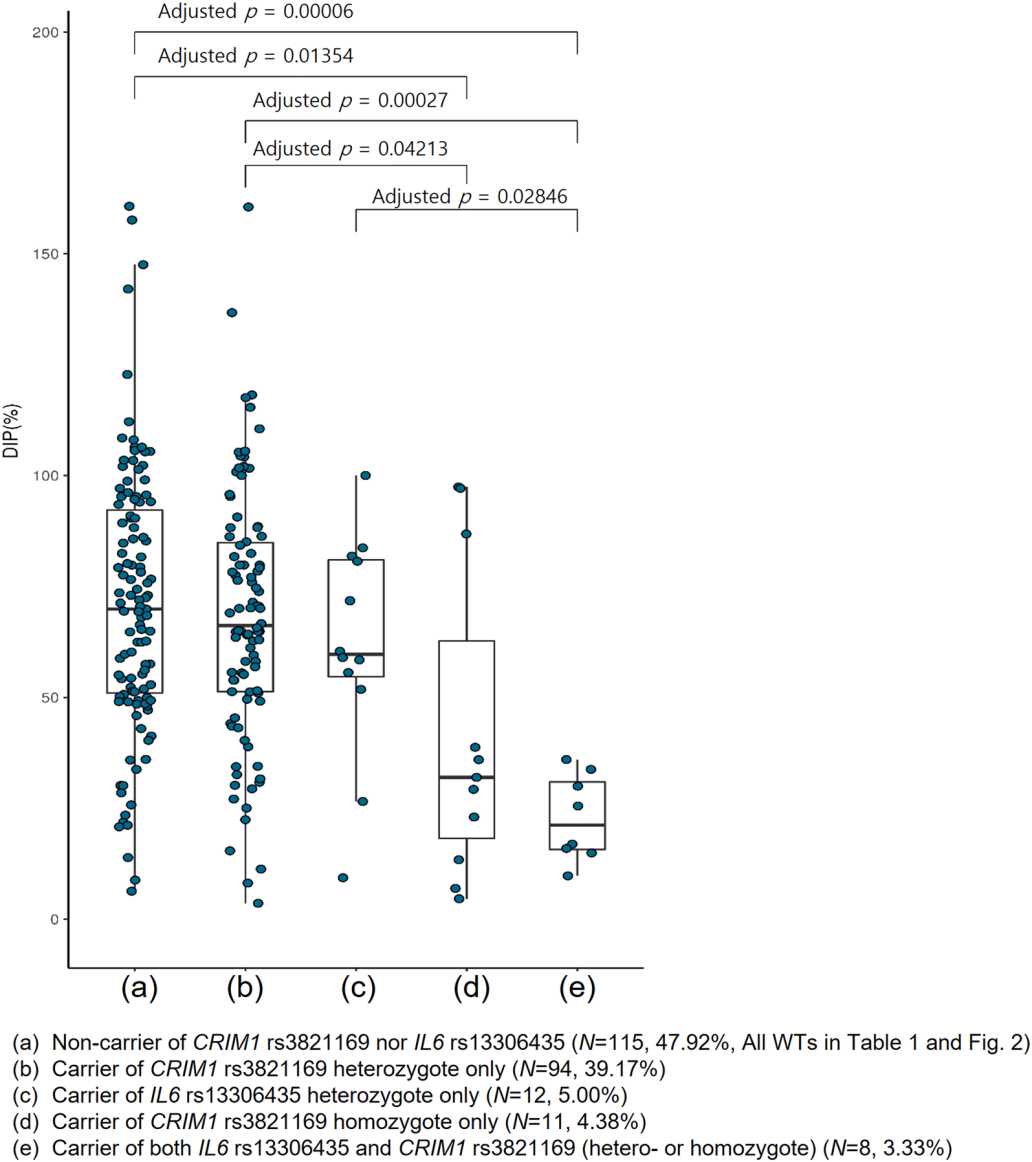
Distribution of the average of the tolerated last-cycle 6-MP DIPs (%) of the *CRIM1* rs3821169 and/or *IL6* rs13306435 variant carrier *vs*. non-carrier subgroups among 240 pediatric patients with ALL presenting both *NUDT15* and *TPMT* WT characterization. Of the 320 pediatric patients with ALL, we excluded 80 carriers presenting either *NUDT15* or *TPMT* variants to obtain 240 subjects with both *NUDT15* and *TPMT* WT. Both (**a**) non-carrier group of *CRIM1* rs3821169 or *IL6* rs13306435 (*N* = 115, 47.92%, i.e., “All WTs” in Table 2 and Fig. 3) and (**b**) carrier group of heterozygous *CRIM1* rs3821169 only (*N* = 94, 39.17%) showed significantly higher thiopurine tolerance than the carrier groups of (**d**) homozygous *CRIM1* rs3821169 (*N* = 11, 4.38%) (adj. *p* < 0.05, posthoc Tukey) and of (**e**) both *IL6* rs13306435 and *CRIM1* rs3821169 (*N* = 8, 3.33%) (adj. *p* < 0.0005, posthoc Tukey) by one-way ANOVA (*p* = 0.0001). (**c**) Carrier group of *IL6* rs13306435 heterozygous variant only (*N* = 12, 5.00%) showed significantly higher thiopurine intolerance than (**c**) that of the hetero/homozygous variant of both *IL6* and *CRIM1* (adj. *p* < 0.05, posthoc Tukey). No carrier of homozygous *IL6* was detected, and only one subject carried both heterozygous *IL6* and homozygous *CRIM1* variants (DIP = 9.7%). Thiopurine intolerance was measured by the last-cycle 6-MP DIP (%) among 240 pediatric patients with ALL presenting both *NUDT15* and *TPMT* WT genes to control their effect on thiopurine intolerance. *p < 0.05 and **p < 0.01, posthoc Tukey test after one-way ANOVA. *ALL* acute lymphoblastic leukemia, *WT* wild-type, *DIP (%) *dose intensity percentage, *6-MP* 6-mercaptopurine.

**Figure 3:**
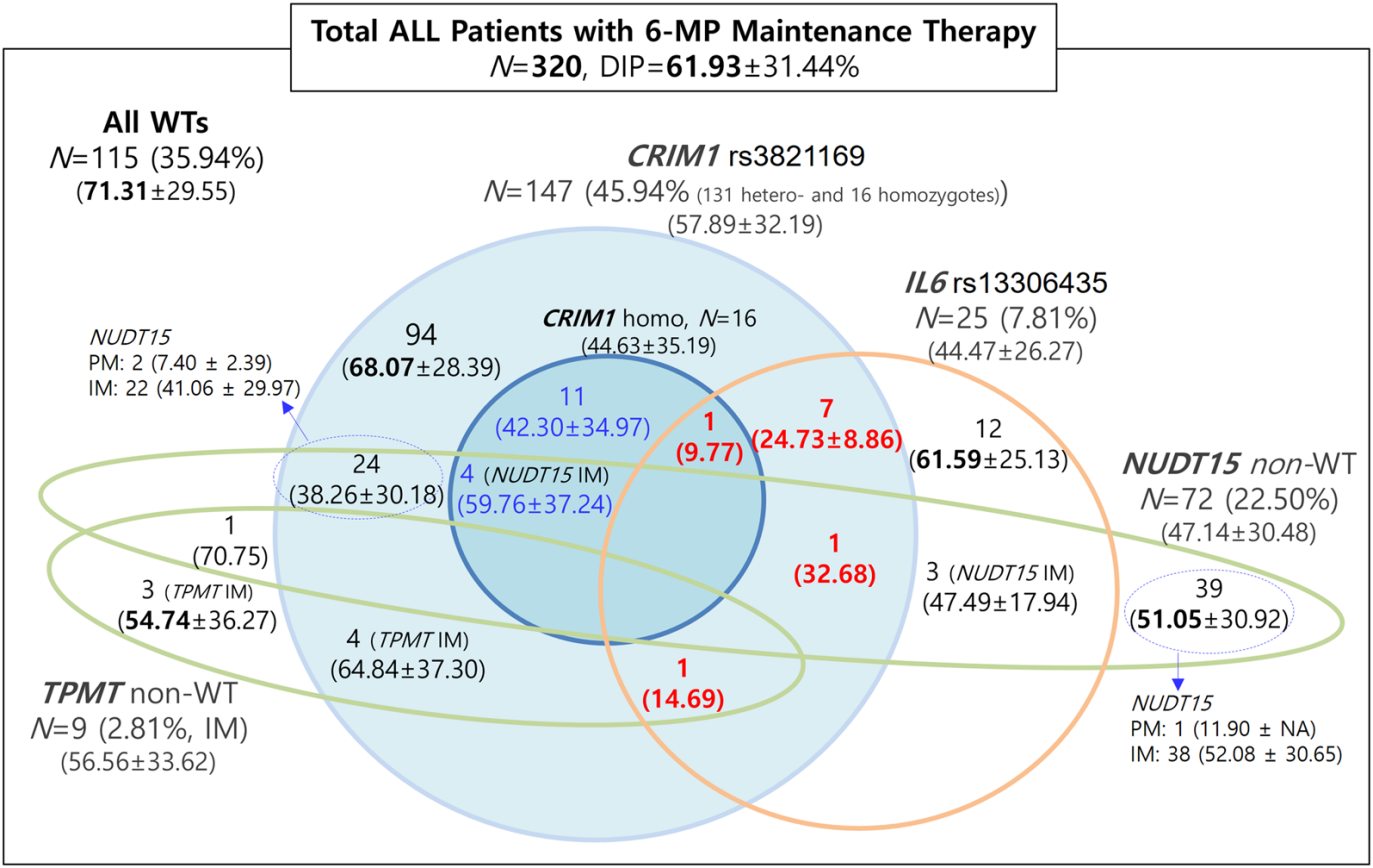
Distribution of the last-cycle 6-MP DIP for pediatric patients with ALL according to *NUDT15*, *TPMT*, *CRIM1,* and *IL6* pharmacogenetic subgroups (N = 320). Green circles depict *NUDT15* and *TPMT* metabolism phenotypes, and blue and orange circles represent *CRIM1* rs3821169 and *IL6* rs13306435 genotype subgroups, respectively. Of the 320 patients, 115 with no pharmacogenetic variants exhibited higher 6-MP DIPs (71.31%) than 72 *NUDT15* (47.14%), 9 *TPMT* (56.56%), 147 *CRIM1* (57.89%), and 25 *IL6* (DIP = 44.47%) non-WTs. Subjects with both *CRIM1* and *IL6* variants (*N* = 10, 3.13%) exhibited the lowest DIPs (9.77–32.68%, in red numbers). Numbers are the number of subjects and 6-MP DIPs (mean ± SD). *ALL *acute lymphoblastic leukemia, *WT* wild-type, *non-WT* non-wild-type (i.e., poor- or intermediate thiopurine-metabolizing sub-groups of *NUDT15* and *TPMT* carriers), *DIP* dose intensity percentage, *SD* standard deviation.

Figure 2 exhibits the distributions of the last-cycle 6-MP DIPs (%) of 115 all WTs (Fig. 2a), carriers of only heterozygous *CRIM1* (*N* = 94, Fig. 2b), carriers of only heterozygous *IL6* (*N* = 12, Fig. 2c), carriers of only homozygous *CRIM1* (*N* = 11, Fig. 2d), and carriers of both *IL6* and *CRIM1* variants (*N* = 8, Fig. 2e). Homozygous *CRIM1* and *IL6* and *CRIM1* groups showed significantly lower 6-MP DIPs (44.6 ± 35.2% and 24.7 ± 8.9%, respectively, Fig. 2d,e) than all WTs and heterozygous *CRIM1* groups (71.3 ± 29.6% and 68.1 ± 28.4%, respectively, Figs 2a,b) by one-way ANOVA (*p* = 0.0001; adj. *p* < 0.05 posthoc Tukey). Furthermore, the *IL6* and *CRIM1* group showed significantly lower 6-MP DIPs (44.6 ± 35.2%) than the heterozygous *IL6* group (61.6 ± 25.1%; adj. *p* < 0.05, posthoc Tukey) (Fig. 2c,e). All 10 patients with both *IL6* and *CRIM1* variants with any *NUDT15* or *TPMT* status (red numbers in Fig. 3) exhibited the lowest DIPs (9.77–32.68%) among all subgroups of the whole PGx groups. Thus, a significant interplay between *IL6* and *CRIM1* in thiopurine intolerance was suggested.

**Table 2:** Inter-ethnic variability of thiopurine toxicity-associated pharmacogenetic variants.

	East Asian (N = 504)	South Asian (N = 489)	European (N = 503)	American (N = 347)	African (N = 661)
***NUDT15***					
NM	390 (77.4%)	421 (86.1%)	498 (99.0%)	312 (89.9%)	658 (99.5%)
IM	80 (15.9%)	62 (12.7%)	2 (0.4%)	27 (7.8%)	1 (0.2%)
PM	8 (1.6%)	3 (0.6%)		2 (0.6%)	
Indeterminate	26 (5.2%)	3 (0.6%)	3 (0.6%)	6 (1.7%)	2 (0.3%)
***TPMT***					
NM	481 (95.4%)	472 (96.5%)	463 (92.0%)	301 (86.7%)	520 (78.7%)
IM	22 (4.4%)	17 (3.5%)	35 (7.0%)	40 (11.5%)	77 (11.6%)
PM				2 (0.6%)	6 (0.9%)
Indeterminate	1 (0.2%)		5 (1.0%)	4 (1.2%)	58 (8.8%)
***IL6*, *CRIM1***					
Both WT	269 (53.4%)	440 (90.0%)	478 (95.0%)	281 (81.0%)	658 (99.5%)
*CRIM1* rs3821169 heterozygote only	177 (35.1%)	46 (9.4%)	9 (1.8%)	13 (3.7%)	1 (0.2%)
*IL6* rs13306435 heterozygote only	14 (2.8%)	3 (0.6%)	15 (3.0%)	48 (13.8%)	2 (0.3%)
*CRIM1* rs3821169 homozygote only	33 (6.5%)	0	0	0	0
*IL6* rs13306435 heterozygote and *CRIM1* rs3821169 hetero- or homozygote	10 (2.0%)	0	0	4 (1.2%)	0
*IL6* rs13306435 homozygote and *CRIM1* rs3821169 heterozygote	1 (0.2%)	0	1 (0.2%)	1 (0.2%)	0

Whole-genome sequences of multiple ethnic groups were obtained from the 1000 Genomes Project (N = 2504). Haplotypes and diplotypes were determined by the CPIC allele-definition tables and molecular phenotypes by the CPIC diplotype-phenotype matching tables.

*NM* normal metabolizer, *IM* intermediate metabolizer, *PM* poor metabolizer, *WT* wild-type.

Notably, it was more clinically relevant to evaluate the magnitude of the actual decrease in 6-MP DIP (%) tolerated by patients than the mere statistical significance affected by the study sample size and biomarker prevalence. Table 1 shows that more than one-quarter of the patients with homozygous *CRIM1* (36.4%) and *IL6* and *CRIM1* (50.0%) tolerated less than 25% of the planned DIP, increasing the risk of thiopurine therapeutic failure. The DIPs of our cohorts were comparable with those of the recommended 6-MP doses published in the current CPIC guideline when *NUDT15* or *TPMT* variants were involved^28^. Furthermore, when we raised the DIP cutoff from 25 to 35%, the proportions of homozygous *CRIM1* and *IL6* and *CRIM1* groups increased to 54.6% (6/11) and 87.5% (7/8), respectively, which far exceeded 38.9% and 33.3% of *NUDT15* (28/72) and *TPMT* (3/9) non-WTs, respectively. Notably, only 6.1% (7/115) and 10.5% (12/115) of all WTs tolerated less than 25% and 35% of the planed DIP (Table 1).

### Inter-ethnic variabilities in carrier frequencies and molecular phenotypes

Both *NUDT15* and *TPMT* show wide inter-ethnic variabilities. Table 2 exhibits inter-ethnic variabilities of the PGx variants and molecular phenotypes of the four thiopurine pharmacogenes computed from among 2504 subjects of the 1000 Genomes Project^9^. *NUDT15* non-WT (i.e., IM or PM) is common in East (22.6%) and South (13.9%) Asians but rare in Europeans and Africans (< 1%). In contrast, *TPMT* non-WT is common in Europeans (8.0%) and Americans (13.3%) but relatively rare in Asians (< 5.0%).

Novel PGx variant, *CRIM1* rs3821169, demonstrates remarkably high minor allele frequency (T = 0.255) and carrier prevalence (43.7%, 220/504) in East Asians. Table 2 also shows that 6.5% of East Asians harbor homozygous *CRIM1* rs3821169 variant, which can hardly be detected in other populations (< 1.0%). In contrast, *IL6* rs13306435 is widely distributed with the highest carrier frequency of 15.0% in Americans and 3.0% among Asian and European populations; It is rare in South Asian and African populations (< 1.0%). The carrier frequencies of both *IL6* and *CRIM1* variants were 2.0% and 1.2% for East Asian and American populations, respectively.

### Single- and multi-gene prediction performances of *IL6* and *CRIM1*

**Figure 4:**
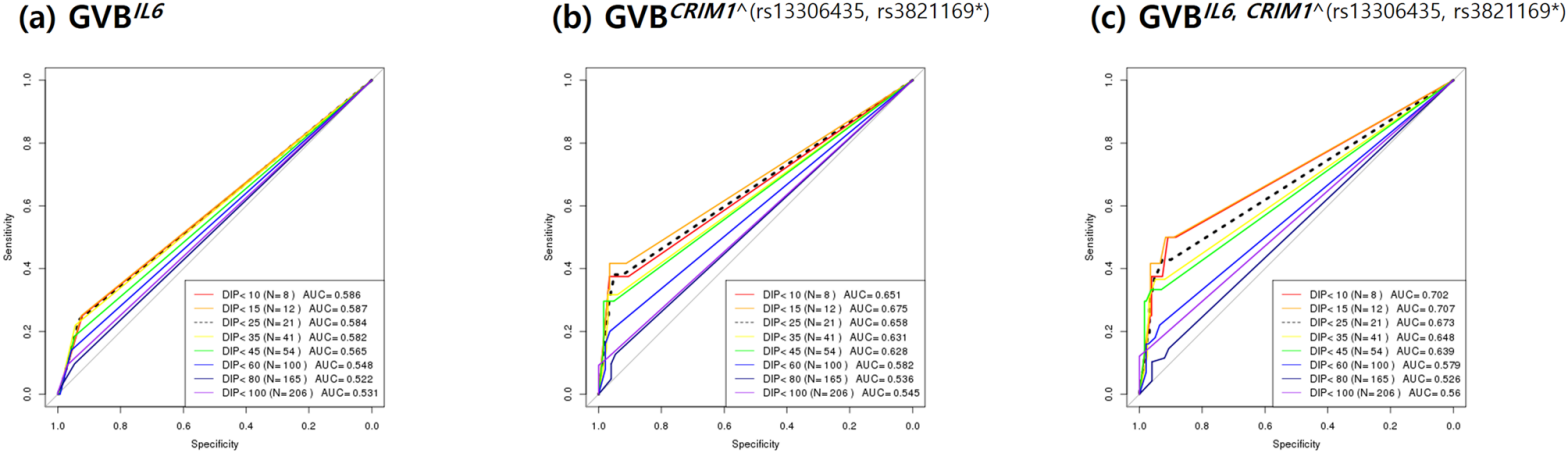
ROC analysis of *IL6*, *CRIM1*, and their combined prediction accuracies for thiopurine intolerance among pediatric patients with ALL presenting wild-type *NUDT15* and *TPMT* genes (N = 240). Single-gene prediction models of (**a**) *IL6* and (**b**) *CRIM1* were highly outperformed by (**c**) the two-gene combined model for predicting thiopurine intolerance at all DIP levels. We excluded 80 subjects from among the total of 320 subjects to control the effects of the well-established *NUDT15* and *TPMT* genes and performed ROC analysis for the 240 subjects with both wild-type *NUDT15* or *TPMT* genes. Prediction accuracies were measured across 8 cutoff levels for the last-cycle 6-MP DIP (%) (≤ 10%, ≤ 15%, ≤ 25%, ≤ 35%, ≤ 45%, ≤ 60%, ≤ 80%, and ≤ 100%) in terms of AUC. *ROC* receiver operating characteristics, *DIP (%)* dose intensity percentage, *AUC* area under the ROC curve, *GVB* gene-wise variant burden; GVB^CRIM1^(rs13306435, rs3821169*)^, GVB of *CRIM1* dependent on *IL6* rs13306435 or *CRIM1* rs3821169 homozygote; 6-MP, 6-mercaptopurine.

We performed ROC analysis of GVB-based single- and multi-gene models to predict the last-cycle 6-MP DIPs (%) using 240 both WTs for *NUDT15* and *TPMT* to control their long-known PGx effects. Figure 4 demonstrates that (b) GVB^*CRIM1*^ outperformed (a) GVB^*IL6*^ in predicting DIPs at all cutoff levels, probably due to the higher variant frequency of *CRIM1* over *IL6* in the study population. Two-gene model GVB^*IL6,CRIM1*^ (Fig. 4c) consistently outperformed each of the single-gene models (GVB^*IL6*^ and GVB^*CRIM1*^) at all cutoffs.

**Figure 5:**
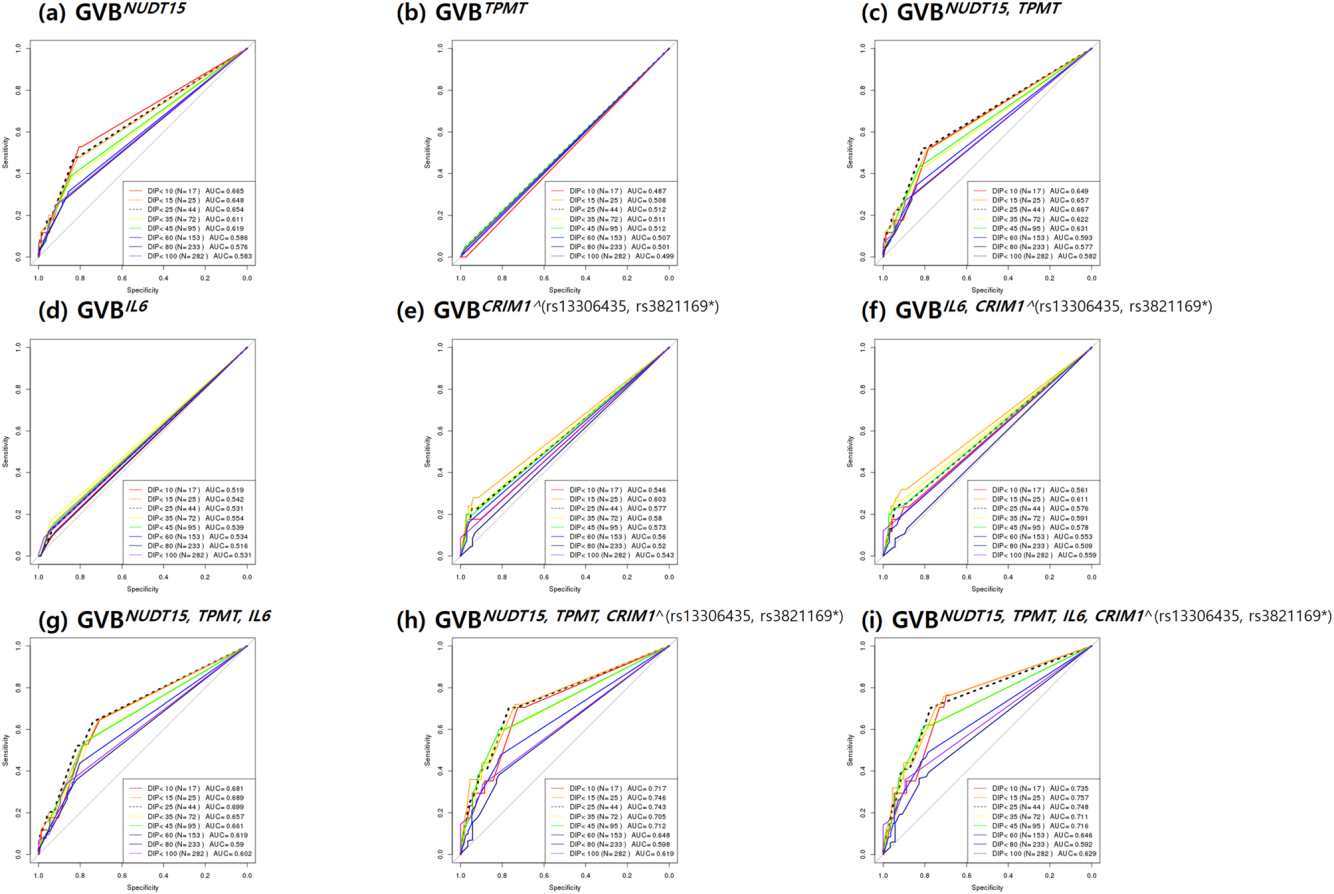
Prediction accuracy profile of single- and multi-gene models for thiopurine intolerance in pediatric patients with ALL (N = 320). Single-gene prediction models of (**a**) *NUDT15* and (**b**) *TPMT* were outperformed by (**c**) the two-gene combined model and those of (**d**) *IL6* and (**e**) *CRIM* by (**f**) the *IL6-CRIM1* combined model. Three-gene models of *NUDT15*, *TPMT*, and (**g**) *IL6* and (**h**) *CRIM1* were outperformed by (**i**) all four-gene combined models. Overall the final (**i**) four-gene combined models outperformed other models for predicting thiopurine intolerance at all DIP levels in pediatric patients with ALL (N = 320). AUCs for predicting the last-cycle 6-MP DIP (%) were measured at 8 cutoff levels (≤ 10%, ≤ 15%, ≤ 25%, ≤ 35%, ≤ 45%, ≤ 60%, ≤ 80%, and ≤ 100%). *ALL* acute lymphoblastic leukemia, *DIP* dose intensity percentage, *AUC* area under the receiver operating characteristics curve, *GVB* gene-wise variant burden.

For a comprehensive evaluation of all PGx interactions among *NUDT15*, *TPMT, IL6*, and *CRIM1*, we performed comprehensive ROC analysis using data of all 320 pediatric patients with ALL (Fig. 5). Among the four single-gene models in Fig. 5a,b,d), GVB^*NUDT15*^ outperformed others at all cutoffs, probably due to the high prevalence of *NUDT15* variants and the strong metabolic impact of on thiopurine toxicity. Two-gene models (Fig. 5c,f) consistently outperformed each of the corresponding single-gene counterparts, i.e., the order of their AUCs was GVB^*NUDT15,TPMT*^ > GVB^*NUT15*^ > GVB^*TPMT*^ and of GVB^*IL6,CRIM1*^ > GVB^*CRIM1*^ > GVB^*IL6*^ at all cutoff levels. Three-gene models created by adding *IL6* or *CRIM1* to the traditional *NUDT15* and *TPMT* model also consistently improved the prediction accuracy (Fig. 5g,h). The final four-gene model in Fig. 5(i) outperformed all other models in predicting DIPs at all cutoff levels. Moreover, it is worth noting that the ROC curves across eight DIP cutoffs in Fig. 5 exhibited ‘dose–response relationships’, i.e., GVB score’s prediction power (measured by AUC) increases as a function of the severity of thiopurine intolerance (measured by DIP). That is, the final four-gene model’s AUC increased as a function of decreasing DIP (%) (i.e., AUC^<15%^ = 0.757, AUC^<25%^ = 0.748, AUC^<35%^ = 0.711, AUC^<45%^ = 0.716, AUC^<60%^ = 0.646, and AUC^<80%^ = 0.592 in a descending order, Fig. 5i).

### Evaluation of the clinical validity and utility of the star allele and GVB methods

We systematically compared the clinical utility as well as clinical validity of traditional star (*) allele-based and GVB-based methods for preventing thiopurine toxicity. Table 3 demonstrates the measures of clinical validity and potential population impact along with the pharmacogenetic association of the different prediction models^14^. Because the designated star alleles for *IL6* or *CRIM1* were not available, star allele-based molecular phenotyping was not applicable for these novel genes.

**Table 3:** Contingency tables for predicting thiopurine intolerance (DIP < 25%) of two-, three-, and four-gene models in pediatric patients with ALL (*N* = 320).

(a)				(c)				(e)			
STAR^***NUDT15***, ***TPMT***^	DIP (%)		Total	GVB^***NUDT15****, ****TPMT****, ****IL6***^	DIP (%)		Total	GVB^***NUDT15****, ****TPMT, CRIM1***^(r13306435, rs3821169^*^)^	DIP (%)		Total
	≤ 25	> 25			≤ 25	> 25			≤ 25	> 25	
PM + IM	**23**	57	80	≤ 0.3	**23**	51	74	≤ 0.3	**31**	64	95
NM	21	**219**	240	> 0.3	21	**225**	246	> 0.3	13	**212**	225
Total	**44**	276	320	Total	**44**	276	320	Total	**44**	276	320

(b)				(d)				(f)			
GVB^***NUDT15****, ****TPMT***^	DIP (%)		Total	GVB^***NUDT15****, ****TPMT****, ****IL6****, *CRIM1^*	DIP (%)		Total	GVB^***NUDT15****, ****TPMT****, ****IL6****, ****CRIM1***^(rs13306435*,* rs3821169^*^)^	DIP (%)		Total
	≤ 25	> 25			≤ 25	> 25			≤ 25	> 25	
≤ 0.3	**23**	53	76	≤ 0.3	**28**	60	88	≤ 0.3	**31**	63	94
> 0.3	21	**223**	244	> 0.3	16	**216**	232	> 0.3	13	**213**	226
Total	**44**	276	320	Total	**44**	276	320	Total	**44**	276	320
(g)											

Prediction models	Sensitivity (%)	Specificity (%)	PPV (%)	NPV (%)	OR	Relative risk	PAF	NNT	NNG		
(a) STAR^***NUDT15****, ****TPMT***^	52.27	79.35	28.75	91.25	4.21	3.286	0.364	5.000	20.000		
(b) GVB^***NUDT15****, ****TPMT***^	52.27	80.80	30.26	91.39	4.61	3.516	0.374	4.618	19.442		
(c) GVB^***NUDT15****, ****TPMT****, ****IL6***^	52.27	**81.52**	31.08	91.46	4.83	3.641	0.379	4.436	19.181		
(d) GVB^***NUDT15****, ****TPMT****, *CRIM1^*	64.64	78.26	31.82	93.10	6.30	4.614	0.498	4.013	14.591		
(e) GVB^***NUDT15****, ****TPMT***, ***CRIM1***^(r13306435, rs3821169^*^)^	70.46	76.81	32.63	94.22	7.90	5.648	0.580	3.724	12.544		
(f) GVB^***NUDT15****, ****TPMT****, ****IL6****, ****CRIM1***^(rs13306435*,* rs3821169^*^)^	**70.46**	77.17	**32.98**	**94.25**	**8.06**	**5.733**	**0.582**	**3.673**	**12.503**		

*ALL *acute lymphoblastic leukemia, *DIP (%)* the last-cycle 6-MP dose intensity percentage, *PAF* population attributable fraction, *PPV* positive predictive value, *NNT* number needed to treat, *NNG* number needed to genotype, *NPV* negative predictive value, *OR* odds ratio, *M* poor metabolizer, *IM* intermediate metabolizer, *STAR*^*NUDT15, TPMT*^ classical star (*) allele-based haplotyping of *NUDT15* and *TPMT* genes according to the CPIC guideline, *GVB* gene-wise variant burden, *GVB*^CRIM1^(rs13306435, rs3821169^*^)^, GVB of *CRIM1* dependent on *IL6* rs13306435 or *CRIM1* rs3821169 homozygote, GVB cutoff value of 0.3 was selected as maximized Youden’s index.

Bold number means True Positive (TP) group, True Negative (TN) group and patients with thiopurine intolerance(DIP<25%). For example, In Table 3a, 23 means TP group and 219 means TN group. 44 indicates that number of patients with thiopurine intolerance(DIP<25%) is 44. Additionally, the most strongest statistical parameter numbers related to clinical validity at the bottom of the table was highlighted in bold. For example, prediction model (g) and (e) showed the most desirable Sensitivity(70.46%) among all prediction models. In a similar aspect, model (f) showed the best effectiveness in number needed to trat (NNT, 3.673) and number needed to genotype (NNG, 12.503).

GVB^*NUDT15,TPMT*^ slightly outperformed STAR^*NUDT15,TPMT*^, the classical star (*) allele-based molecular phenotyping (Table 3a,b). Three-gene models (i.e., GVB^*NUDT15,TPMT,IL6*^ and GVB^NUDT15,TPMT,CRIM1*^ also outperformed the two-gene models (Table 3c,d). The four-gene interplay model, GVB^*NUDT15,TPMT,IL6,CRIM1*^(*CRIM1,IL6*)^, presented the best performance for all the eight measures of clinical validity and potential population impact (except specificity) (marked in bold numbers in Table 3g).

The addition of *IL6* and *CRIM1* to create the final four-gene model by integrating both common and rare alleles markedly improved PAF from 0.36 to 0.58 as well as RR (3.29–5.73) and OR (4.21–8.06). PAF is the proportion of events attributed to the PGx risk factor or the maximum percentage of cases that can be prevented if individuals who test positive for the PGx variants receive different treatments.

In the group of all the patients with 6-MP toxicities (DIP < 25%), we could expect eight patients more with the GVB-based model than with the traditional star (*) allele-based method [23 patients in Table 3a *vs*. 31 patients in Table 3f]. NNG is the number of patients that must be genotyped to prevent one patient from experiencing an adverse event. The NNG of 20 and an NNT of 5 of the traditional STAR^*NUDT15,TPMT*^ showed that 5 out of every 20 patients genotyped would be found to have positive test results for genotyping and would need alternative treatment to prevent toxicity related 6-MP intolerance in one patient (DIP < 25%). Adding *IL6* and *CRIM1* to the traditional *NUDT15* and *TPMT* testing to create GVB^*NUDT15,TPMT,IL6,CRIM1*^(*CRIM1,IL6*)^ may require only 12.5 patients (37.5% improvement of NNG) to be genotyped to return 3.7 test-positive patients (26.0% improvement of NNT) receiving alternative treatment to prevent adverse event in one patient (Table 3g).

### Ethics approval and consent to participate

Informed written consent was obtained from all subjects, and the study was approved by the ethics committees of Asan Medical Center, Seoul National University Hospital, and Samsung Medical Center.

## Discussion

In the present study, the interplay between *IL6* and *CRIM1* variants in thiopurine intolerance due to hematological toxicity was investigated in 320 pediatric patients with ALL.

*IL6* has been known to modulate hematopoiesis and neutrophil trafficking, especially possessing a role in anti-apoptosis^29^. In patients with osteomyelitis, *IL6* was correlated with longer neutrophil survival apart from other cytokines; this anti-apoptotic effect was blocked using anti-IL6 antibodies and reversed with anti-*IL6*^30^. According to a recent study on chronic hepatitis C infection, the expression of anti-apoptotic genes was increased following in vitro IL6 treatment, with a considerable downregulation in T cell inhibitory receptors and caspase-3, thus indicating the promising nature of *IL6* in enhancing lymphocyte effector functions^31^. These anti-apoptotic roles seemed to be secondary to the effects of IL6 trans-signaling, such as the inhibition of the chemokines CXCL1, CXCL8 and CX3CL1 and the promotion of the chemokines CXCL5, CXCL6, and CCL2; however, the suppression of *IL6* might result in blood cytopenia. The occurrence of neutropenia as an adverse effect of the *IL6* inhibitor, tocilizumab, could also demonstrate the relationship between *IL6* and neutrophil survival. *IL6* mobilizes neutrophils into the circulating pool from the marginated pool comprising the lymph nodes and the spleen. Therefore, neutropenia due to a lack of IL6 induced by tocilizumab may indicate that IL6 has a critical role in enriching circulating neutrophils^32^.

*IL6* variant, rs13306435, is located in exon 5 of the *IL6* gene. The T>A variation of rs13306435 has an altered amino acid, from Asp to Glu. The T allele of 13306435 is reportedly associated with increased expression and plasma levels of *IL6*^33^. In this regard, patients with a heterozygous variant of rs13306435 might have decreased expression and plasma levels of *IL6* compared with the patients with the WT characterization, resulting in reduced *IL6* effects on neutrophils.

*CRIM1* is a cell-surface transmembrane protein that resembles developmentally important proteins which are known to interact with bone morphogenetic proteins (BMPs). A role of *CRIM1* in drug resistance has been suggested by previous studies^34,35^ revealing that the level of mRNA expression of *CRIM1* is high in resistant leukemic cells. This affects the levels of BMPs, suggesting that *CRIM1* regulates the growth and differentiation of hematopoietic cells. The rs3821169 heterozygous cases revealed lower mRNA expression levels than the WT cases, which indicated that subjects carrying this variant might display drug-sensitive responsiveness^8^. Although we could not clarify the detailed mechanism underlying the interplay between *IL6* and the *CRIM1* variant, the presence of a negative feedback loop between IL6 and the BMP pathway was reported, in which increased levels of IL6 induced BMP pathway activities resulting in the suppression of IL6^36^. Based on our findings, it can be suggested that the interplay between *IL6* and *CRIM1* in thiopurine intolerance due to hematological toxicity may represent a pharmacodynamic effect leading to an adverse reaction, while the well-known *NUDT15* and *TPMT* are pharmacokinetic enzymes for metabolizing thiopurines.

The present study presented several limitations that need to be acknowledged, including the possible confounding effects from concomitant medications (methotrexate or vincristine) and the absence of serum level measurements of drugs or metabolites. Moreover, not all patients with thiopurine toxicity were explained by pharmacogenetic analysis. Seven (6.1%) of the 115 all-WT patients experienced thiopurine toxicity. Supplementary Table S2 lists further candidate variants determined by analyzing the all WTs (*N* = 115, *p* < 0.05 by one-sided Student’s *t* test). Of the three carriers of *FSIP2* rs191083003, two (66.7%) exhibited DIP < 25% (8.82, 21.88, and 48.54%, *N* = 3). We observed one more *FSIP2* rs191083003 carrier in the homozygous-*CRIM1* group, who exhibited the lowest DIP of 6.94% within the entire ALL cohort (*N* = 320). The low frequency (1.25%, 4/320) of *FSIP2* rs191083003 prohibited any conclusion, necessitating further elucidation. Overall, the interplay between *IL6* and *CRIM1* and the well-known *NUDT15* and *TPMT* improved PAF from 36.4 to 58.2% by considering PGx variants only in an East Asian cohort of pediatric ALL (*N* = 320). The quantitative analytical approach employed in the present study could be applied to other ethnic groups for further discover and evaluate thiopurine-related pharmacogenomics.

Reportedly, Americans present the highest allele frequency of *IL6* rs13306435 (A = 0.078) among all ethnic groups (Global A = 0.020, the 1000 Genomes Project, Phase 3^9^). This high inter-ethnic variability may partially explain why rs13306435 has not yet been identified as a biomarker for thopurine intolerance. Current research is mostly biased towards Europeans^37^. *NUDT15* rs116855232 variant, which was recently discovered in the Korean population as a strong predictor of thopurine toxicity^3^, shows the highest allele frequency in East Asians (T = 0.095) among all ethnic groups (Global T = 0.040). Pharmacogenes, by definition, unlike pathogenic disease genes, do not have an overt phenotype unless exposed to drugs. The absence of detrimental phenotypic effect attributed to phrmacogenes may have permitted wide inter-ethnic variability and/or diversity across different ethnic groups under various evolutionary selection pressures.

For pediatric ALL, the CPIC guideline for thiopurine treatment is based on the star (*) allele-based haplotypes, with designated molecular phenotypes of *NUDT15* and *TPMT*^6,7^. However, CPIC does not provide general standard rules for combining multi-gene interactions of the categorically classified star-alleles. Novel genes like *IL6* and *CRIM1* have no designated star alleles nor molecular phenotypes. The quantitative GVB method has benefits over categorical star allele-based approaches. GVB quantitates single- or multi-gene PGx burden of common, rare, and novel variants into a single score to provide a comprehensive framework for further PGx discovery and evaluation of many gene interactions. A conventional single variant-based association test of rare variants requires an infeasible magnitude of sample sizes^38^; however, approaches that aggregate common, rare, and novel variants jointly will substantially reduce the required effective sample sizes^39^. In contrast to a traditional haplotyping-based method, GVB assigns a gene-level score for each pharmacogene without using population data, enabling an unbiased PGx approach, especially for under-investigated subpopulations.

## Conclusion

In summary, our results suggest an independent and/or additive effect of the interplay between *IL6* rs13306435 and *CRIM1* rs3821169 on thiopurine intolerance attributed to hematological toxicity in pediatric ALL*.*

## Supplementary Information

Supplementary Information 1.

## Data Availability

All data generated or analyzed during this study are included in this article. If any additional information is required, it may be obtained by request from the corresponding author.

## Abbreviations

ALL: Acute lymphoblastic leukemia
DIP: Dose intensity percentage
GVB: Gene-wise Variant Burden
CPIC: Clinical Pharmacogenetics Implementation Consortium
PGx: Pharmacogenetic
PM: Poor metabolizer
IM: Intermediate metabolizer
NM: Normal metabolizer
WT: Wild-type
WES: Whole-exome sequencing
RR: Relative risk
PAF: Population attributable fraction
NNT: Number needed to treat
NNG: Number needed to genotype
6-MP: 6-Mercaptopurine
SNUH: Seoul National University Hospital
AMC: Asan Medical Center
SMC: Samsung Seoul Medical Center
CCG: Children’s Cancer Group
ANC: Absolute neutrophil count
SIFT: Sorting intolerant from tolerant
AUCs: Areas under the ROC curves
OR: Odds ratio
IL-6R: Interleukin 6 receptor
CRIM1: Cysteine-Rich Transmembrane BMP Regulator 1
BMPs: Bone morphogenetic proteins
