# Supplementary material for: Interplay between IL6 and CRIM1 in thiopurine intolerance due to hematological toxicity in leukemic patients with wild-type NUDT15 and TPMT
Source: Sci Rep. 2021 May 6;11:9676. doi: 10.1038/s41598-021-88963-5 (PMC8102572; doi:10.1038/s41598-021-88963-5)
Supplement: Supplementary file 1 — Supplementary Information 1. [file 41598_2021_88963_MOESM1_ESM.docx]

**Interplay between IL6 and CRIM1 on thiopurine intolerance due to hematological toxicity in leukemic patients with wild-type *NUDT15* and *TPMT***

Hyery Kim^1†^, Seungwon You^2†^, Yoomi Park^2^, Jung Yoon Choi^3,4^, Youngeun Ma^5^, Kyung Tak Hong^3^, Kyung-Nam Koh^1^, Sunmin Yun^2^, Kye Hwa Lee^2,6^, Hee Young Shin^3^, Suehyun Lee^2,7^, Keon Hee Yoo^8^, Ho Joon Im^1*^, Hyoung Jin Kang^3,4*^, Ju Han Kim^2*^

**Supplementary Table S1. Candidate list which passed gene-wise, variant-wise multiple covariate linear regression. Three candidate variants were investigated with essential information, such as chromosome, gene name, 1000 genome project allele frequency, and consequence.**

**1KG, 1000 genome project. AF, Allele Frequency.**

| CHR | BP | REF | ALT | Gene Name | 1KG  AF | 1KG  AFR_AF | 1KG  AMR_AF | 1KG  EAS_AF | 1KG  EUR_AF | 1KG  SAS_AF | Consequence | SIFT | CADD | dbSNP |
| --- | --- | --- | --- | --- | --- | --- | --- | --- | --- | --- | --- | --- | --- | --- |
| 7 | 22771039 | T | A | *IL6* | 0.0204 | 0.0015 | 0.0778 | 0.0258 | 0.0169 | 0.0031 | missense_variant | 0.41 | 3.534 | rs13306435 |
| 12 | 57883741 | C | T | *MARS* | 0.0064 | 0 | 0 | 0.0308 | 0 | 0.001 | synonymous_variant | 0.01 | 14.08 | rs117101415 |
| 12 | 57906592 | G | A | *MARS* | 0.0064 | 0 | 0 | 0.0308 | 0 | 0.001 | synonymous_variant | 1 | 17.87 | rs117633211 |

CHR, Chromosome; BP, intermediate metabolizer; REF, reference allele; ALT, alternative allele; 1KG, 1000 genome project; AF, allele frequency; AFR_AF, African allele frequency; AMR_AF, American allele frequency; EAS_AF, East Asian allele frequency; EUR_AF, European allele frequency; SAS_AF, South Asian allele frequency; SIFT, sorting intolerant from tolerant; CADD, combined annotation dependent depletion.

**Supplementary Table S2. Candidate variants among 115 patients with ALL from the all wild types (for *NUDT15*, *TPMT*, *IL6* and *CRIM1*) group**

| Variant rsID | Gene | Carrier (%) | Hetero/ Homozygote | Carrier DIP  (mean±SD) | Non-carrier DIP  (mean±SD) | SIFT | CADD | ExAC | P^*^ |
| --- | --- | --- | --- | --- | --- | --- | --- | --- | --- |
| rs191083003 | *FSIP2* | 2.6% | 3 / 0 | 26.4±20.3 | 72.5±28.9 | 0.01 | 26.7 | 0.003 | 0.0260 |
| rs12587478 | *KLHL33* | 5.2% | 5 / 1 | 44.9±22.4 | 72.8±29.3 | 0 | 25.0 | 0.040 | 0.0136 |
| rs200982819 | *SLC15A3* | 6.1% | 6 / 1 | 50.6±21.1 | 72.7±29.6 | 0 | 29.7 | 0.028 | 0.0164 |
| rs67877771 | *IQCG* | 32.3% | 35 / 2 | 61.9±25.4 | 75.8±30.5 | 0.04 | 26.2 | 0.215 | 0.0059 |
| rs61758536 | *SPAG8* | 13.9% | 16 / 0 | 60.5±22.5 | 73.1±30.3 | 0 | 26.0 | 0.052 | 0.0297 |
| rs34337292 | *OR9Q2* | 26.1% | 29 / 1 | 63.1±24.8 | 74.2±30.7 | 0 | 25.9 | 0.068 | 0.0263 |

^*^*p*-value by one-sided t-test. ALL, acute lymphoblastic leukemia; DIP, dose intensity percentage; SIFT, sorting intolerant from tolerant; CADD, combined annotation dependent depletion; ExAC, exome aggregation consortium; SD, standard deviation.
